# Improving Precision of Proximity Ligation Assay by Amplified Single Molecule Detection

**DOI:** 10.1371/journal.pone.0069813

**Published:** 2013-07-16

**Authors:** Rongqin Ke, Rachel Yuan Nong, Simon Fredriksson, Ulf Landegren, Mats Nilsson

**Affiliations:** 1 Science for Life Laboratory, Department of Biochemistry and Biophysics, Stockholm University, Solna, Sweden; 2 Department of Immunology, Genetics and Pathology, the Rudbeck Laboratory, Science for Life Laboratory, Uppsala University, Uppsala, Sweden; 3 Olink Bioscience, Uppsala, Sweden; Northeastern University, United States of America

## Abstract

Proximity ligation assay (PLA) has been proven to be a robust protein detection method. The technique is characterized by high sensitivity and specificity, but the assay precision is probably limited by the PCR readout. To investigate this potential limitation and to improve precision, we developed a digital proximity ligation assay for protein measurement in fluids based on amplified single molecule detection. The assay showed significant improvements in precision, and thereby also detection sensitivity, over the conventional real-time PCR readout.

## Introduction

Developments in protein biomarker assays promise to offer greatly improved opportunities for disease diagnostics and prognostics [1]. In this regard, analytical tools of improved performance will be required to accelerate discovery of disease-related protein biomarkers as well as using these in clinical practise. To date, a number of ultrasensitive protein detection assays have been developed [2], [3], [4], [5], [6], [7], [8]. Among these, PLA has the unique virtue of allowing the configuration of assays that depend on recogntion of target molecules by sets of two, three, or more antibodies, followed by readout via DNA reporter molecules. Accordingly, PLA has been shown to offer high sensitivity and specificity, broad dynamic range, and suitability for multiplex analyses and to analyze complex target molecules [9], [10], [11], [12], [13], [14], [15], [16].

In PLA, detection of proteins by oligonucleotide-modified antibodies gives rise to amplifiable reporter DNA strands via a DNA ligation step. Typically, a pair of PLA probes - antibodies equipped with DNA strands - binds the same target protein molecule, bringing the PLA probes into close proximity so that the DNA strands of the PLA probes are covalently joined through enzymatic DNA ligation. The linear DNA reporter strands that form are then amplified and quantified, usually by real-time PCR, where the abundance of DNA reporters reflects the original concentrations of target proteins in the interrogated samples. Real-time PCR serves as a robust, specific and sensitive readout for PLA.

PLA has been applied in several different formats and for different purposes, including solution-phase (homogenous) and solid-phase (heterogenous) assay formats for detection of single or multiple proteins in complex body fluids [9], [10], [11], [12], [13], [14], [15], [16]. Compared to solution-phase PLA, which normally uses 1–2 µl sample, solid-phase PLA (spPLA) offers the possibility to investigate larger sample volumes, something that can be valuable for detection of target molecules are present at low concentration in plasma. Moreover, the solid-phase format of the assay imposes a requirement that target proteins are bound by three antibodies, augmenting the specificity of the assays.

Readout of PLA reactions via real-time PCR has the drawback of limited quantitative precision inherent to exponential PCR amplification [17]. The intra-assay coefficient of variations (CVs) of spPLA using real-time PCR as readout were found to range from below 10% to greater than 30% [9], which would normally be unacceptable for clinical diagnostics. Recently, several approaches have been presented to improve the precision of DNA analyses by detecting and counting individual molecules, allowing quantification only limited by the poisson noise, which can be brought to low levels provided sufficient numbers of molecules are counted. Counting of DNA molecules is possible using next generation sequencing or via digital PCR where the reactions are compartmentalized in microfabricated structures [18] or in aqueous droplets in emulsions [19]under conditions where only a fraction of the compartments include even a single target molecule. We have demonstrated that the process of rolling circle amplification (RCA) provides a convenient and effcient means of enumerating single DNA strands by a process we refer to as amplified single molecule detection (ASMD), provided the DNA strands have been circularized [20]. The RCA products self-assemble into micrometer-sized individual DNA balls that can be labelled and digitally quantified, without the need for compartmentalization under limiting dilution conditions. In previous work we have used next generation sequencing to digitally quantify spPLA-products, however, in this first study only marginal improvement of the quantitative precision was demonstrated [10], perhaps due to variation introduced by the multiple steps for sequencing library preparation and a PCR based DNA enrichment step. In order to meet the critical need for high precision in protein detection, we now demonstrate digital recording of reaction products from PLA, allowing individual amplification products to be recorded digitally [20].

## Results and Discussion

**Figure 1:**
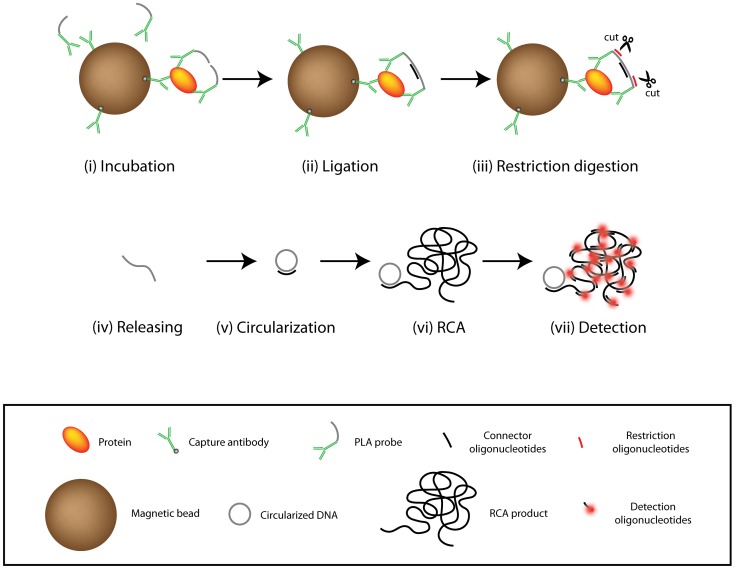
Schematic illustration of protein detection by using PLA with digital ASMD readout.

The principle of digital PLA is illustrated in Figure 1. Target protein molecules present in the samples are captured by antibodies immobilized on magnetic beads, followed by binding of the captured proteins by pairs of PLA probes (Fig. 1 i). After removal of excess PLA probes and unbound sample components through washes, DNA strands on the PLA probes are joined by DNA ligation, assisted by a connector oligonucleotide that serves as a ligation template (Fig. 1 ii). Next, two oligonucleotides are added to guide restriction digestion of DNA strands connecting pairs of antibodies (Fig. 1 iii). The released DNA strands are then circularized in the presence of a DNA template via a second DNA ligation reaction (Fig. 1 iv–v). The circularized reporter DNA molecules are finally amplified by RCA, primed by the same oligonucleotide that first served to template the circularization reaction (Fig. 1 vi). RCA produces long, single stranded DNA molecules, each composed of tandem repeats of the complement of the circularized DNA molecule. These long DNA strands of several tens of thousands nucleotides form random coils with an average diameter around 1 µm, which after hybridization with fluorescence dye labeled detection probes become brightly fluorescent (Fig. 1 vii). The fluorescent RCA products are counted using a dedicated microfluidic ASMD instrument [13].

**Figure 2:**
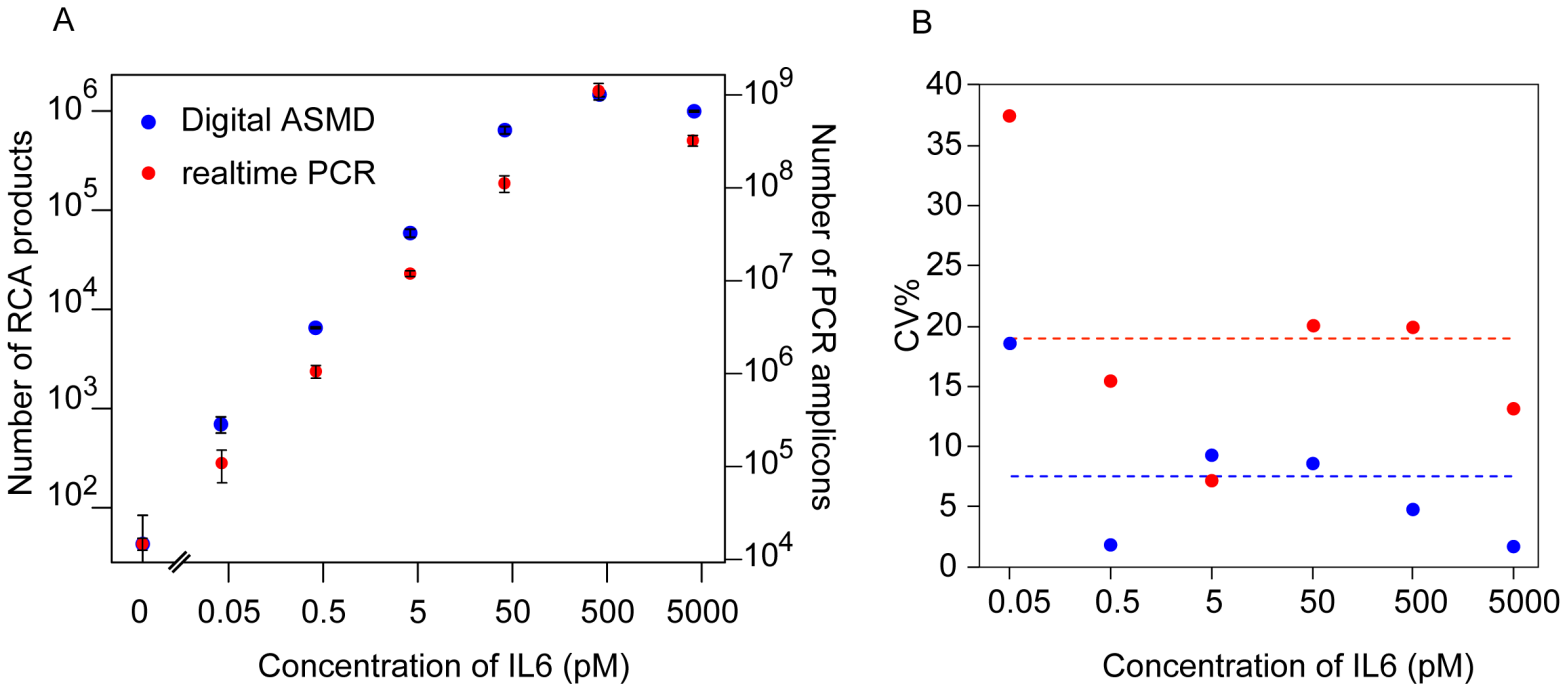
(A) Standard curves for IL6 detection by PLA followed by digital ASMD readout (blue dots) or realtime PCR based readout (red dots). Error bar = ±1SD. (B) Comparison of the CV% between the two readout strategies (blue dots: digital ASMD readout; red dots: realtime PCR readout).

**Figure 3:**
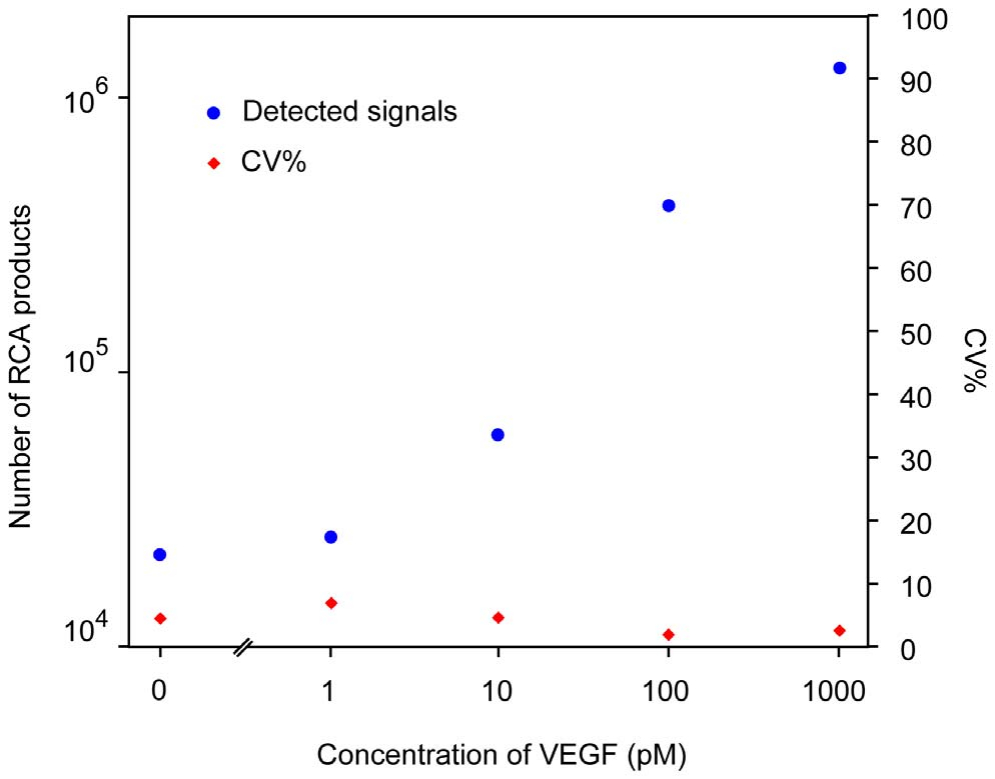
Detection of VEGF by PLA digital ASMD readout. Blue dots: detected signal; Diamond symbol: CV%.

To determine the performance of the approach for measuring proteins in liquid phase, we applied the assay to analyze the cytokine interleukin 6 (IL6). IL6 was diluted in buffer over a 6 log concentration range, with the lowest concentration representing physiological levels of IL6 in blood of 50 fM (1 pg/ml) (Figure 2A). The digital quantification of PLA products resulted in an extrapolated LOD (limit of detection, defined as the concentration at RCA counts 2×SD over background) of approximately 5 fM (0.1 pg/ml) and average CVs ∼ 7% across a dynamic range of 5 logs. PCR readout performed in parallel (Figure 2B), resulted in 1.5 fold higher, that is less sensitive LOD of approximately 9 fM, and the average CVs for the PCR readout was ∼18% over a comparable dynamic range. ASMD thus provided at least two fold improved precision compared to the PCR-based readout. A similar quantitative precision was observed in detection of the cytokine vascular epithelial growth factor (VEGF) in buffer (Figure 3). We observed that ASMD measurement of solid phase PLA reaction products exhibited greater variation than seen when RCA products were diluted, where the average CV was ∼3% if more than 1000 RCA products were counted for each dilution (Figure S1). The additional variation from solid phase PLA may be explained by contributions from imprecise liquid handling during PLA and variable efficiency of capture of target proteins and their recognition by PLA probes. Automation of magnetic bead handling, or using homogenous forms of PLA [11] may further decrease variation introduced by the immunoassay part of the method.

In conclusion, we have developed a method for digital readout of spPLA. We demonstrate that PLA with digital readout, where ligation products from spPLA are counted via ASMD, is a precise and sensitive procedure for detecting analytes like plasma proteins. In this approach, each ligation product is amplified to become one RCA product, composed of hundreds of complements of the ligation product, easily detectable by ASMD. Therefore, this approach offers digital detection of protein markers in liquid phase, providing improved precision as well as better sensitivity compared to previous protocols. Previously, spPLA products have been amplified either by bulk PCR or using the so called circle-to-circle amplification prior to digital quantification [10], [13]. By contrast, the ligation products in the current protocol were directly enumerated after individual amplification by RCA, eliminating sources of imprecision introduced by the amplification step. The current approach preserves other advantages of PLA, such as high specificity, wide dynamic range, and suitability for multiplex analysis. In comparison to other single molecule amplification approaches, such as digital PCR [18], or emulsion PCR [19], in which conditions are sought to amplify single DNA molecules in individual reaction wells or aqueous droplets in oil, our approach is simple and easy to implement through RCA of circularized reaction products, using standard heating blocks and incubators. The reaction products were then allowed to pass through a microfluidic channel where individual fluorescent amplification were detected and counted.

The improved precision of digital PLA renders the PLA technique even more suitable for implementation in clinical diagnostics. Our work establishes counting of RCA products as a powerful general strategy to go from the nanometer scale of molecular detection to easily recorded micrometer scale objects for sensitive and precise detection of DNA reporter strands.

## Materials and Methods

### Oligonucleotides, Proteins, Antibodies and PLA Probes

Antibodies and recombinant proteins were purchased from R&D systems (Table S1).

Oligonucleotides were purchased from IDT (Table S2).

PLA probes with antibodies conjugated to DNA oligonucleotides (Table S2) were prepared using the protocol described previously [10]. All oligonucleotides were purchased from Integrated DNA Technologies.

### Solid-phase PLA

Solid phase PLA was performed as previously described [13], except that capture antibodies and conjugates were diluted to 1 nM during incubation with target proteins. Recombinant protein IL6 was diluted in a concentration series of which 45 µl were mixed with 5 µl of beads and conjugates mix. Incubations of antibodies and targets were carried out at room temperature for 2–3 hours. Each solid phase PLA reaction was performed at a single concentration of protein and all measurements were performed in hexaplicate. Ligation products from three replicate assays were amplified and detected by real time PCR following the protocol described previously [10], whereas ligation products from the other three replicate assays were quantified via ASMD as described below.

### DNA Release by Digestion, Circularization, and RCA

After proximity ligation, magnetic beads, some of them carrying PLA immune-complexes, were washed three times with 1×PBS supplemented with 0.05% Tween-20 (PBS-T). To release the newly formed DNA strands from the solid phase, a restriction digestion mixture (20 µl) was added that contained 1×phi29 polymerase buffer (Fermentas), 2 µg/µl BSA, 0.05 µM of each restriction digestion oligonucleotide (I and II), 0.5 U/µl MboI (Fermentas), 0.5 U/µl AluI (New England Biolabs, NEB) and 0.01 U/µl uracil-DNA glycosylase (UNG) (Fermentas). The reactions were incubated at 37°C for 10 min, followed by 65°C for 5 min to inactivate the enzymes. The supernatants, containing released DNA strands, were transferred to new tubes with 5 µl ligation mixture consisting of 4×Ampligase buffer (80 mM Tris-HCl pH 8.3, 100 mM KCl, 40 mM MgCl_2_, 20 mM β-nicotinamide adenine dinucleotide (NAD), 0.4% Triton X-100), 0.2 µM ligation template and 0.5 U/µl Ampligase (Epicentre Biotech). After vortexing thoroughly, the ligation reactions were incubated at 55°C for 20 min. RCA was initiated by adding 5 µl of RCA mix containing 1×phi29 polymerase buffer (Fermentas), 2 µg/µl BSA, 500 µM of dNTPs (Fermentas) and 0.6 U/µl of phi29 polymerase (Fermentas). After incubation at 37°C for 1 h, the RCA reaction was terminated by heating to 65°C for 1 min.

### Preparation of RCA Products from Circularized Padlock Probes

First, 20 nM padlock probes were circularized in a 50 µl ligation mixture containing 1×phi29 polymerase buffer, 1 mM ATP (Fermentas), 60 nM ligation templates, and 0.02 U/µl T4 DNA ligase (Fermentas). The reaction was carried out at 37°C for 15 min. The resulting DNA circles were then diluted in 1×PBS to desired concentrations. Afterwards, RCA was carried out as described above to generate RCA products from the circular DNA molecules. RCA products were serially diluted and divided into three tubes for triplicate detection.

### Detection of RCA Products and Data Analysis

After RCA, the products generated by RCA were mixed with detection buffer to a final volume of 100 µl. The final concentrations of each composition was: 5 nM detection probes, 1 M NaCl, 2 mM EDTA, 20 mM Tris-HCl (pH 8.0), 0.1% Tween-20. Hybridization was performed at 80°C for 1 min and 65°C for 10 min. Finally, RCA products with fluorescent detection probes were counted in a microfluidic flow channel with laser excited fluorescence detection in an instrument described in Göransson *et al* [13]. Twenty images of each sample were collected and analyzed. The number of detected objects in these images was summed up as the final result for each sample.

The limit of detection was defined as the protein concentration that gave rise to a signal two standard deviations above the mean of the background noise. Signals are measured as numbers of RCA products counted via ASMD, or estimated numbers of starting amplification templates for PCR readout. Numbers of amplification templates for PCR readout (N) were calculated from Ct values using the equation N = 2×(40–Ct). This formula assumes that 40 is the Ct value obtained for a single starting amplification template in PCR. The coefficient of variation was calculated for each dilution point using the equation CV% = standard deviationx100/mean.

## Supporting Information

Figure S1 **Detection of dilutions of RCA products by ASMD quantification.** Blue dots: detected signal; Diamond symbol: CV%.
(TIF)

Table S1 **Antibodies and recombinant proteins.**
(DOCX)

Table S2 **Oligonucleotides.**
(DOCX)
