# Supplementary figures and images for: Improving Precision of Proximity Ligation Assay by Amplified Single Molecule Detection
Source: PLoS One. 2013 Jul 16;8(7):e69813. doi: 10.1371/journal.pone.0069813 (PMC3713053; doi:10.1371/journal.pone.0069813)

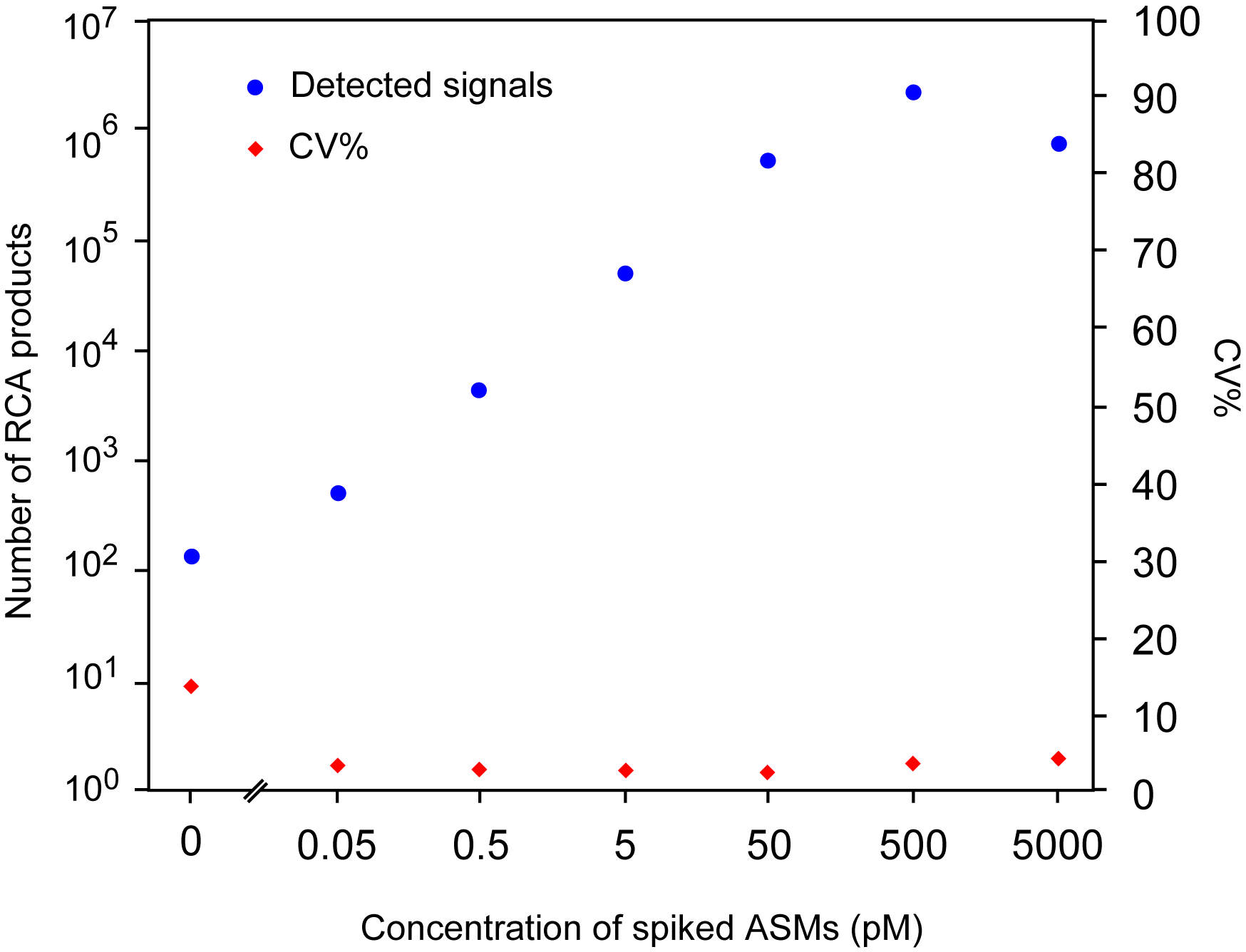

Supplement: Figure S1 — Detection of dilutions of RCA products by ASMD quantification. Blue dots: detected signal; Diamond symbol: CV%. (TIF) [file pone.0069813.s001.tif]
