# Supplementary material for: Improving Precision of Proximity Ligation Assay by Amplified Single Molecule Detection
Source: PLoS One. 2013 Jul 16;8(7):e69813. doi: 10.1371/journal.pone.0069813 (PMC3713053; doi:10.1371/journal.pone.0069813)
Supplement: Table S1 — Antibodies and recombinant proteins. (DOCX) [file pone.0069813.s002.docx]

**Table S1** Antibodies and recombinant proteins

| Name | Catalog |
| --- | --- |
| Recombinant Human IL-6 | 206-IL-010 |
| Human IL-6 Affinity Purified Polyclonal Ab, Goat IgG | AF-206-NA |
| Human IL-6 Biotinylated Affinity Purified PAb, Goat IgG | BAF206 |
| Recombinant Human VEGF 165 | 293-VE-010 |
| Human VEGF 165 Affinity Purified Polyclonal Ab, Goat IgG | AF-293-NA |
| Human VEGF 165 Biotinylated Affinity Purified PAb, Goat IgG | BAF293 |
