# Supplementary material for: Improving Precision of Proximity Ligation Assay by Amplified Single Molecule Detection
Source: PLoS One. 2013 Jul 16;8(7):e69813. doi: 10.1371/journal.pone.0069813 (PMC3713053; doi:10.1371/journal.pone.0069813)
Supplement: Table S2 — Oligonucleotides. (DOCX) [file pone.0069813.s003.docx]

**Table S2** Oligonucleotides

| Name | Sequences (5’to 3’) | 5' modification | 3' modification |
| --- | --- | --- | --- |
| PLA arm 1 | AGTGAGCTAGACTTATTGCGTCACGATGAGACTGGATGAA | Thiol | - |
| PLA arm 2 | TCACGGTAGCATAAGGTGCAGGCGATCCAATTATCAGTAC | Phosphate | Thiol |
| Connector | CUACCGUGAUUCAUCCAG | - | - |
| PCR primer 1 | AGTGAGCTAGACTTATTGCG | - | - |
| PCR primer 2 | GTACTGATAATTGGATCGCC | - | - |
| Restriction digestion oligonucleotides I | GTCTAGCTCACT | - | - |
| Restriction digestion oligonucleotides II | GATAATTGGATCGCCT | - | - |
| Ligation template (for PLA assay) | ACGCAATAAGTCTAGGCCTGCACCTTATGC | - | - |
| Padlock probe | ATGGTCAGGCTTGGTTTGTGGATAGTGTCTTACACGAAGAGTGTACCGACCTCAGTAAGTCTCCTAGCTCGGTGAACTAGTCTGTATCAAC | Phosphate | - |
| Ligation template (for padlock probe) | CTCTCTCTCTCTCTAAACCAAGCCTGACCATGTTGATACAGACT | - | - |
|  |  |  |  |
|  |  |  |  |
